# Sociodemographic factors, health seeking behaviors, reproductive history, and knowledge of cervical screening among women in Swaziland

**DOI:** 10.1186/s13027-020-00282-y

**Published:** 2020-03-05

**Authors:** Ibironke O. Aina, Smruti M. Raul, Luz A. Padilla, Simangele Mthethwa-Hleta, Peter O. Preko, Pauline E. Jolly

**Affiliations:** 1 Department of Epidemiology, School of Public Health, University of Alabama at Birmingham, 1655 University Boulevard, Birmingham, AL 35294-0022 USA; 2 Ministry of Health and Social Welfare, 2nd Floor Ministry of Justice & Constitutional Affairs Building, Mhalambanyatsi Road, Mbabane, Swaziland; 3 Care and Treatment Lead for the President’s Emergency Plan for AIDS Relief (PEPFAR), Jubela Street; Kent Rock, Mbabane, Swaziland

**Keywords:** Swaziland, Cervical screening, Sociodemographic factors, Health-seeking behaviors, Reproductive history, Knowledge of cervical cancer, Visual inspection with acetic acid

## Abstract

**Background:**

Cervical cancer is the leading cause of cancer among women in Swaziland; however, a low rate of cervical screening in this population has led to high rates of morbidity and mortality from cervical cancer.

**Objective:**

To identify factors associated with lack of cervical screening among women in Swaziland.

**Methods:**

A cross-sectional study was conducted among 300 women aged 18–69 years attending clinics in three regions of Swaziland from May to August of 2014. An investigator-administered questionnaire was used to collect data on socioeconomic factors, health-seeking behaviors, reproductive history, and cervical screening history and knowledge from the women.

**Results:**

Adjusted multivariable logistic regression analysis revealed that women < 30 years of age were less likely to receive a cervical exam compared to women ≥30 years of age (Odds Ratio 0.06, 95% Confidence Interval 0.01–0.67). Women who had a tertiary education were almost 6 times more likely to receive a cervical screening (OR 5.83, 95% CI 1.11–30.50). Women who said that they did not know when to receive cervical screening were 73% less likely to have a cervical exam (OR 0.27, 95% CI 0.01–0.74).

**Conclusions:**

Younger age, lower educational level, and lack of knowledge about when to receive a cervical screening affected whether women obtained a cervical screening. This indicates the need for educating women, particularly younger women, about the importance of cervical examinations. Addressing these barriers to screening should lead to a decrease in cervical lesions and cancer, especially in this high HIV-positive population.

## Background

Cervical cancer (CC) is the fourth most common cancer among women worldwide and the number one cancer among women in sub-Saharan Africa [1]. Over 85% of the global burden of CC occurs in resource-limited countries [1]. There are 530,000 new CC cases and 275,000 deaths related to CC annually. Most of the morbidity and mortality from CC occurs because of low cervical screening rates (19% on average) among women in developing countries [2]. Many women do not visit health care facilities until they are in the advanced stages of CC.

Almost all cases of CC are caused by oncogenic strains of the Human Papillomavirus (HPV) [3–6]. In women with healthy immune systems, most HPV infections are transient and are eventually cleared from the body. There are an estimated 380 new cases of CC annually in Swaziland [7]. A recent study from Swaziland showed that the prevalence of high-risk-HPV (hr-HPV) infection was high and significantly associated with HIV among sexually active women [8]. The estimated overall prevalence of hr-HPV was 46.2% and decreased with increasing age. The overall HIV prevalence among the women was 42.7% and HIV-positive women were 5 times more likely to be infected with hr-HPV and to have multiple group hr-HPV infections [8]. The overall hr-HPV/HIV co-infection was 24.4% and was significantly higher among younger women [8]. Thus, the risk of cervical lesions among HIV-positive women in Swaziland is 4 to 5 times higher than among HIV-negative women [9]. Swaziland has the highest HIV prevalence rate (27.4%) in the world, with women of reproductive age (15–49 years) making up more than half of all HIV infections in the country [10]. The HIV prevalence among pregnant women in Swaziland is estimated at 39.2% [11]. With regard to factors associated with cervical lesions, we found that women who had ≥2 lifetime sexual partners and women with a history of sexually transmitted infections (STIs) were three and two times more likely, respectively, to have cervical lesions compared to women with one lifetime partner and no previous STIs [9].

Other studies have identified factors such as smoking, sexual debut before age 16, age < 40 years, CD4 count < 650 cells, ≥5 abortions, and other vaginal wall abnormalities to be associated with cervical lesions or invasive CC [12–14]. Furthermore, factors such as knowledge and attitudes towards cervical screening, as well as availability and accessibility of services, will determine the extent to which women will participate in screening and other health services [15].

Due to the lack of technical, infrastructural, financial, and human resources needed for cytology-based screening in many developing countries, visual inspection with acetic acid (VIA) is the cervical screening method most commonly used in low-income countries, including Swaziland [16]. During cervical screening, physicians apply acetic acid to the cervix to determine if there are any lesions. VIA is considered a more efficient option due to rapid exam results that allows for immediate treatment when necessary [16, 17].

In this study, we investigated sociodemographic factors, health-seeking behaviors, knowledge of CC screening and accessibility in relation to cervical screening history among a sample of women, including HIV-positive women, attending health clinics in Swaziland. It is essential to understand women’s health-seeking behaviors and knowledge of cervical screening so that appropriate interventions can be implemented to increase screening uptake, which could lead to a decrease in CC cases.

## Methods

A cross-sectional study was conducted among 300 women (150 HIV-positive and 150 HIV-negative) 18–69 years of age. The women were recruited from three hospitals in Swaziland that were able to perform cryotherapy and VIA, namely Mbabane Hospital in the Hhohho Region, the Raleigh Fitkin Memorial (RFM) Hospital in the Manzini Region, and the Hlatikulu Hospital in the Shiselweni Region. HIV-positive women were recruited from antiretroviral therapy (ART) clinics and HIV treatment and care (HTC) sites at outpatient departments (OPDs) of the hospitals. The comparison group was women who tested HIV-negative at the OPDs.

Clinic staff informed the women of the study and asked if they would be willing to participate. Women who expressed interest in participating were introduced to the study staff who told them about the purpose and procedures of the study. Participants were guaranteed full confidentiality and participation was voluntary. The women were told that they could refuse participation and withdraw from the study at any time. The women read the informed consent form and were encouraged to ask questions prior to signing the form. After participants provided signed consent, an interviewer-administered questionnaire was used to collect information on: 1) Sociodemographic factors, HIV status, and cervical screening (age, marital status, income, education, employment status, occupation); 2) CC knowledge and use of health care services (family history of CC, frequency of use of health care facilities); 3) Knowledge and perceptions regarding cervical screening (knowing which test to have, knowing when to be screened, worry about screening results, cost of screening, screening test is painful, embarrassing and/or uncomfortable); and 4) Reproductive history and sexual practices (parity, breastfeeding, contraception use, sexual partners, condom use, history of STIs). Each participant was assigned a unique identification (ID) number that was placed on their questionnaire; no personal identifying information was written on the questionnaire. To help ensure confidentiality, the interviews were conducted in private hospital rooms. Once the interview was complete, clinic staff used VIA to screen the participants for cervical lesions.

### Data analysis

Data were analyzed for 297 women, three of the women were excluded from the analysis due to unknown HIV status; 87 of the 297 women (29.3%) previously received a cervical exam and 210 had not. Sociodemographic, sexual, and reproductive history variables were stratified according to whether the women reported that they had a previous cervical exam. Descriptive statistics (frequencies, percentages, means, and standard deviations) were performed on all variables and used to summarize the data for the study groups. Differences among the variables between the groups were compared using Chi-square and t-test analysis. Logistic regression was used to run two regression models. The first regression model was adjusted for age, as there was a significant age difference in cervical screening among the women. The second model was a fully adjusted multivariable model that included all variables with a *p*-value of < 0.1 in the bivariate analysis to explore the association with having had a cervical exam. Statistical Analytical Software 9.4 (SAS Institute, Cary, NC) was used for the analysis and all statistical tests of a two-sided *p*-value of < 0.5 were considered significant.

## Results

A significant difference was observed between women who previously had a cervical exam and those who did not for participants’ age (*p* = 0.003; Table 1). Marginal differences were observed for education level, employment status, alcohol consumption, and VIA test results (Table 1). Significant differences were observed for whether participants reported hearing of VIA (*p* = 0.006) and their use of healthcare services (*p* = 0.014) according to previous cervical screening (Table 2). Analysis of the data examining knowledge and perception of participants toward cervical screening showed a significant difference for participants’ self-reporting not being aware of which tests to receive (*p* <  0.001), and not knowing when to be screened (*p* <  0.001;Table 3). Table 4 shows the analysis between reproductive history and sexual practices by previous cervical exam. None of the variables was statistically different.

**Table 1:** Sociodemographic factors and HIV status and having had a cervical screening

	Previous Cervical Exam			
	Yes	No	Total	*p*-value
	*N* = 87	*N* = 210	*N* = 297	
	*N* (%)	*N* (%)	*N* (%)	
Age				
< 30	21 (24.1)	88 (41.9)	109 (36.7)	0.0032
≥ 30	66 (75.9)	122 (58.1)	188 (63.3)	
Region				
Hhohho	32 (36.8)	79 (38.3)	111 (37.9)	0.2852
Lubombo	4 (4.6)	4 (1.9)	8 (2.7)	
Manzini	43 (49.4)	91 (44.2)	134 (45.7)	
Shiselweni	8 (9.2)	32 (15.5)	40 (13.7)	
Marital Status				
Married/Cohabiting	40 (46)	90 (42.9)	130 (43.8)	0.6222
Widowed/Single/Separated	47 (54)	120 (57.1)	167 (56.2)	
Monthly income (1 US dollar = 14 Swaziland Lilangeni)				
< E500 or None	29 (33.3)	84 (40.2)	113 (38.2)	0.4345
E500–1200	23 (26.4)	56 (26.8)	79 (26.7)	
> E1201	35 (40.2)	69 (33)	104 (35.1)	
Education level				
Primary/No school	17 (19.5)	50 (23.8)	67 (22.6)	0.0865
Secondary	46 (52.9)	126 (60)	172 (57.9)	
Tertiary	24 (27.6)	34 (16.2)	58 (19.5)	
Employment status				
Employed/Self-employed or Partially	59 (67.8)	115 (55.8)	174 (59.4)	0.0541
Unemployed/Student	28 (32.2)	91 (44.2)	119 (40.6)	
Occupation				
Laborer	24 (35.8)	47 (36.2)	71 (36)	0.9932
Skilled worker/Clerical	26 (38.8)	51 (39.2)	77 (39.1)	
Professional	17 (25.4)	32 (24.6)	49 (24.9)	
Smoking status				
Never smoked	83 (95.4)	204 (97.1)	287 (96.6)	0.4619
Former/current smoker	4 (4.6)	6 (2.9)	10 (3.4)	
Drink alcohol?				
Yes	11 (13.1)	14 (6.7)	25 (8.5)	0.0875
No	73 (86.9)	195 (93.3)	268 (91.5)	
HIV status				
Positive	34 (41.5)	77 (41)	111 (41.1)	0.9381
Negative	48 (58.5)	111 (59)	159 (58.9)	
VIA Results				
Positive	15 (18.7)	20 (10.6)	35 (13.1)	0.0794
Negative	65 (81.3)	168 (89.4)	233 (96.9)

**Table 2:** Cervical cancer knowledge and use of care services by having had a cervical exam

	Previous Cervical Exam			
	Yes	No	Total	*p*-value
	*N* = 87	*N* = 210	*N* = 297	
	*N* (%)	*N* (%)	*N* (%)	
Has anyone in your family had cervical cancer?				0.6537
Yes	10 (11.5)	21 (10)	31 (10.4)	
No	75 (86.2)	180 (85.7)	255 (85.9)	
Don’t know	2 (2.3)	9 (4.3)	11 (3.7)	
Do you believe cervical cancer can be prevented?				0.1329
Yes	74 (85.1)	157 (74.8)	231 (77.8)	
No	2 (2.3)	7 (3.3)	9 (3.0)	
Don’t know	11 (12.6)	46 (21.9)	57 (19.2)	
How often should a woman have a PAP smear?				0.5597
No need to have a PAP smear	1 (1.6)	1 (0.6)	2 (0.8)	
Every year	53 (82.8)	150 (88.2)	203 (86.7)	
≥ every 2 years	7 (10.9)	16 (9.4)	23 (9.8)	
Other (i.e. Once a month)	1 (1.6)	1 (0.6)	2 (0.8)	
Have you ever heard of VIA				0.0063
Yes	29 (33.3)	35 (16.7)	64 (21.5)	
No	58 (66.7)	174 (82.9)	232 (78.1)	
Would you be interested in getting screened by VIA				0.1803
Yes	85 (97.7)	208 (99.5)	293 (99)	
No	2 (2.3)	1 (0.5)	3 (1)	
From which of the following sources do you obtain most of your knowledge about health?				0.0832
Your parents	5 (5.9)	28 (13.7)	33 (11.4)	
Your friends	7 (8.2)	10 (4.9)	17 (5.9)	
The media	41 (48.2)	76 (37.1)	117 (40.3)	
Your sexual partner	8 (9.4)	15 (7.3)	23 (7.9)	
Other (HCW, school, family)	24 (28.2)	76 (37.1)	100 (34.5)	
What has been your general approach towards using health care services?				0.0144
Only use whenever I am sick	36 (54.5)	108 (63.2)	144 (60.8)	
Visit yearly for checkup	28 (42.4)	45 (26.3)	73 (30.8)	
Hardly ever use	1 (1.5)	18 (10.5)	19 (8.0)	
Are you more likely to use health care facilities or herbal or local remedy				0.5329
Healthcare facilities	85 (98.8)	209 (99.5)	294 (99.3)	
Herbal Medicine	1 (1.2)	1 (0.5)	2 (0.7)

**Table 3:** Knowledge and perceptions towards cervical screening among women by previous screening

	Yes	No	Total	*p*-value
	*N* = 87	*N* = 210	*N* = 297	
	*N* (%)	*N* (%)	*N* (%)	
Did not know which tests I should have				0.0007
Yes	36 (41.4)	132 (62.9)	168 (56.6)	
No	51 (58.6)	78 (37.1)	129 (43.4)	
Doctor has never recommended screening				0.0938
Yes	49 (56.3)	140 (66.7)	189 (63.6)	
No	38 (43.7)	70 (33.3)	108 (36.4)	
Did not know when to have screening				< 0.0001
Yes	42 (48.3)	158 (75.2)	200 (67.3)	
No	45 (51.7)	52 (27.8)	97 (32.7)	
No one known talked about screening				0.2174
Yes	45 (51.7)	125 (59.5)	170 (57.2)	
No	42 (48.3)	85 (40.5)	127 (42.8)	
Worried about tests finding cancer				0.7395
Yes	33 (37.9)	75 (35.9)	108 (36.5)	
No	54 (62.1)	134 (64.1)	188 (63.5)	
Cancer screening tests cost too much				0.2406
Yes	14 (16.1)	46 (22.0)	60 (20.3)	
No	73 (83.9)	163 (78.0)	236 (79.7)	
Cancer screening tests are painful				0.1420
Yes	36 (41.4)	106 (50.7)	142 (48.0)	
No	51 (58.6)	103 (49.3)	154 (52.0)	
Cancer screening tests are embarrassing and/or uncomfortable				0.1040
Yes	26 (29.9)	44 (20.9)	70 (23.6)	
No	61 (70.1)	166 (79.1)	227 (76.4)	
You don’t think you need testing				0.1823
Yes	23 (26.4)	72 (34.3)	95 (32.0)	
No	64 (73.6)	138 (65.7)	202 (68.0)	
You forget to schedule the tests				0.7652
Yes	25 (28.7)	64 (30.5)	89 (30.0)	
No	62 (71.3)	146 (69.5)	208 (70.0)	
Takes too long to get an appointment				0.8735
Yes	36 (41.4)	89 (42.4)	125 (42.1)	
No	51 (58.6)	121 (57.6)	172 (57.9)	
Did not have time for cancer screening tests				0.5691
Yes	45 (51.7)	101 (48.1)	146 (49.2)	
No	42 (48.3)	109 (51.9)	151 (50.8)

**Table 4:** Reproductive history and sexual practices by having had a cervical exam

	Previous Cervical Exam			
	Yes	No	Total	*p*-value
	*N* = 87	*N* = 210	*N* = 297	
	*N* (%)	*N* (%)	*N* (%)	
How many children do you have?	2.32 ± 2.24	2.05 ± 1.66	2.13 ± 1.84	0.2652
Age at first child				0.7699
≤ 17	32 (36.8)	74 (35.2)	106 (35.7)	
18–23	46 (52.9)	108 (51.4)	154 (51.8)	
≥ 24	9 (10.3)	28 (13.3)	37 (12.5)	
How many of your children did you breastfeed?	2.47 ± 1.31	2.13 ± 1.53	2.23 ± 1.47	0.0913
Are all your children from the same partner?				0.3304
Yes	32 (43.2)	84 (48)	116 (46.6)	
No	29 (39.2)	52 (29.7)	81 (32.5)	
Only 1 child	13 (17.6)	39 (22.3)	52 (20.9)	
Have you ever had an abortion				0.0574
Yes	4 (4.6)	2 (1)	6 (2)	
No	83 (95.4)	206 (99)	289 (98)	
Age at first sexual intercourse				0.9176
≤ 17	33 (37.9)	85 (40.5)	118 (39.7)	
18–23	49 (56.3)	113 (53.8)	162 (54.6)	
≥ 24	5 (5.7)	12 (5.7)	17 (5.7)	
Number of lifetime sexual partners				0.5056
1	20 (23.0)	56 (26.7)	76 (25.6)	
≥ 2	67 (77.0)	154 (73.3)	221 (74.4)	
Condom use				0.7277
Do not use condoms	18 (20.7)	42 (20.1)	60 (20.3)	
Sometimes/Most times/Non-Regular partner only	46 (52.9)	120 (57.4)	166 (56.1)	
History of any of the following STIs				0.1245
Warts, gonorrhea, syphilis, herpes	28 (33.3)	50 (24.4)	78 (27.0)	
None	56 (66.7)	155 (75.6)	211 (73.0)	

Table 5 shows the Odds Ratio (OR) and 95% Confidence Interval (CI) of the age-adjusted and fully adjusted logistic regression models for sociodemographic factors, use of health care services, knowledge of cervical screening, reproductive variables of participants, and history of cervical screening. In the fully adjusted model, previous cervical screening among the women varied by age groups. Participants who were < 30 years of age were 94% less likely to have received a cervical screening than those ≥30 years of age (OR 0.06; 95% CI 0.01–0.67). Participants with a tertiary education were six times more likely to have received a cervical screening than those with lower educational levels (OR 5.83; 95% CI 1.11–30.50). Participants who reported that they did not know when to be screened were 73% less likely to have a cervical exam in the fully adjusted model (OR 0.27; 95% CI 0.01–0.74). Tertiary education and not knowing when to be screened were also significantly different in the age-adjusted model (OR 2.25; 95 95% CI 1.04–4.87 and OR 0.30; 95% CI 0.17–0.50, respectively).

**Table 5:** Regression model for sociodemographic, sexual history, and reproductive practices of having had a cervical screening

	Age-Adjusted OR§ (95%confidence interval)	Fully Adjusted OR (95%confidence interval)
Age		
< 30		0.06 (0.01–0.67)
≥ 30		Referent
Education level		
Primary/No school	Referent	Referent
Secondary	1.13 (0.59–2.17)	1.48 (0.43–5.17)
Tertiary	2.25 (1.04–4.87)	5.83 (1.11–30.50)
Employment status		
Employed/Self-employed or Partially	1.39 (0.80–2.40)	0.61 (0.21–1.74)
Unemployed/Student	Referent	Referent
Drink alcohol?		
Yes	Referent	Referent
No	0.43 (0.18–1.01)	0.65 (0.12–3.68)
VIA Results		
Positive	Referent	Referent
Negative	0.51 (0.24–1.07)	0.34 (0.08–1.38)
From which of the following sources do you obtain most of your health knowledge?		
Your parents	Referent	Referent
Your friends	2.88 (0.72–11.51)	3.87 (0.24–62.42)
The media	2.27 (0.79–6.54)	1.22 (0.14–10.74)
Your sexual partner	2.46 (0.67–9.09)	8.78 (0.66–115.91)
^a^Other	1.35 (0.45–3.99)	1.56 (0.18–13.93)
What has been your general approach towards using health care services?		
Only use whenever I am sick & Hardly ever use	Referent	Referent
Visit yearly for checkup	1.97 (1.07–3.63)	1.87 (0.74–4.72)
Has any of the following ever prevented you from being tested for cervical cancer?		
Not knowing which tests I should have		
Yes	0.39 (0.23–0.65)	0.54 (0.22–1.37)
No	Referent	Referent
Doctor has never recommended screening		
Yes	0.61 (0.36–1.03)	1.36 (0.50–3.65)
No	Referent	Referent
Didn’t know when to have screening		
Yes	0.30 (0.17–0.50)	0.27 (0.01–0.74)
No	Referent	Referent
Have you ever heard of VIA		
Yes	2.61 (1.45–4.70)	1.78 (0.47–6.75)
No	Referent	Referent
How many of your children did you breastfeed?	1.13 (0.94–1.36)	
Have you ever had an abortion		
Yes	5.06 (0.88–29.12)	2.60 (0.24–28.82)
No	Referent	Referent

^a^Other = Health-care worker, school, family

Additionally, in the age-adjusted model, there was a significant difference by participants’ report of their approach towards using health care services. Participants who reported yearly visits to the clinic were twice as likely to have a history of cervical screening compared to those who reported hardly ever visiting the clinic or visiting only when sick (OR 1.97; 95% CI 1.07–3.63). Participants who reported having heard of VIA were 2.6 times more likely to have been screened previously than those who had not heard of VIA (OR 2.61; 95% CI 1.45–4.70).

## Discussion

In the fully adjusted model, we observed that age was a significant factor in history of cervical screening. Participants < 30 years of age were 94% less likely to be screened. This finding is similar to that of a study on CC screening in Kenya which found that women 35–49 years of age were more likely to be screened than women 15–24 years of age [18].

The likelihood of screening among older women may be greater because they have had a longer time to visit health facilities for pregnancy, postnatal care, or other health reasons. This finding has serious implications for the health of younger women living with HIV since they are more likely to develop persistent HPV and pre-invasive cervical lesions [10]. This is especially a concern in Swaziland where the prevalence of HIV is very high among women and contributes to high rates of CC. HIV-positive women have a higher chance of developing CC as much as 10 years before HIV-negative women [19]. This finding highlights the importance of conducting educational and screening interventions for CC among women but especially among younger sexually active women.

We found that women who reported that they did not know when to receive cervical screening were 73% less likely to be screened. Lack of knowledge about CC, benefits of screening to identify lesions early, and effectiveness of treatment for prevention of invasive disease is a major barrier to cervical screening. A study from Swaziland reported that 46.5% of women could not correctly name at least one symptom of CC [20]. Almost 60% of participants in this study had misconceptions about the risk factors for CC and named factors such as witchcraft, abortion, and birth control [20]. Other studies conducted in sub-Saharan Africa report that lack of knowledge was related to low CC screening among women [18, 21–23]. A study conducted in Kenya found that cervical screening was higher among women who had more media exposure, higher household income, were employed, and visited a health facility in the past 12 months [18]. Another study from Kenya found that knowledge of CC risk factors was significantly associated with employment status, possibly suggesting that women may become more aware of health matters such as CC screening through their employment [21]. This lack of knowledge contributes to the women’s misunderstanding of CC leading many women to believe that they are not as susceptible and/or at minimal risk for the disease. To address the lack of CC knowledge among patients, healthcare professionals need to communicate information about CC in a manner that allows patients to ask questions and voice their concerns.

We found that participants with tertiary education were almost six times more likely to receive cervical screening than women with primary or no schooling. This finding is similar to a previous study conducted in Nigeria which found that almost 50% of women in the study lacked knowledge of CC screening services and that lack of awareness varied significantly with their level of education [23]. A study from Kenya also found that Pap smear screenings were higher among communities that had higher proportions of women with higher education [18].

## Limitations

This study has limitations that must be considered in interpreting the results. A main limitation is the cross-sectional study design that does not allow for determination of temporal relationships or determination of causality. The small sample size is another limitation that might have prevented significant findings for variables other than those found significant in the study. Since the study sample was drawn from patients attending healthcare facilities, the results are not generalizable to women who did not visit healthcare facilities. Further, the sample may not be fully representative of women attending clinics in Swaziland. However, we recruited women from the main hospitals in three of the four regions of Swaziland. Finally, the data were self-reported and may be subject to social desirability bias.

Regardless of the limitations, this study identified factors such as age, education, and knowledge of when to obtain cervical screening as important factors influencing whether a woman had previous cervical screening. Identifying these barriers to cervical screening is important in Swaziland because of the very high HIV prevalence among women and high rate of CC. VIA is the cervical screening method most commonly used in Swaziland, however, 78% of women in this study reported that had not previously heard of VIA. The proportion of women who reported that they had heard of VIA and were screened was twice that of women who said they had not previously heard of VIA. Thus, it is crucial for programs to focus on providing CC education and ensuring that women with less education are knowledgeable about CC. Future prevention and intervention strategies should focus on sociodemographic factors, health-seeking behaviors, and knowledge of CC in order to encourage women in Swaziland to seek cervical screening.

## Data Availability

The datasets used and analyzed during the current study are available from the corresponding author upon reasonable request.

## Abbreviations

ART: Antiretroviral therapy
CC: Cervical Cancer
CI: Confidence interval
HPV: Human Papillomavirus
hr-HPV: High risk Human Papillomavirus
HTC: HIV treatment and care
OR: Odds ratio
STI: Sexually transmitted infections
VIA: Visual inspection with acetic acid
